# Shade Delayed Flowering Phenology and Decreased Reproductive Growth of *Medicago sativa* L.

**DOI:** 10.3389/fpls.2022.835380

**Published:** 2022-06-02

**Authors:** Fengfei Qin, Yixin Shen, Zhihua Li, Hui Qu, Jinxia Feng, Lingna Kong, Gele Teri, Haoming Luan, Zhiling Cao

**Affiliations:** ^1^College of Agro-grassland Science, Nanjing Agricultural University, Nanjing, China; ^2^Institute of Grassland Research, Chinese Academy of Agricultural Sciences, Hohhot, China

**Keywords:** *Medicago sativa* L., weak light, flowering phenology, pollen viability, stigma receptivity, fruiting

## Abstract

Alfalfa (*Medicago sativa* L.) is an important forage in intercropping or rotation ecosystem, and shading is the principal limiting factor for its growth under the crop or forest. Agronomic studies showed that shading would systematically reduce the biomass of alfalfa. However, little is known about the reproduction of alfalfa under shading conditions. In order to study the effect of shading on the reproductive characteristics of alfalfa, two alfalfa cultivars (“Victoria” and “Eureka”) were used to study the effect of shading levels (full light, 56.4% shade, and 78.7% shade) on alfalfa flowering phenology, pollen viability, stigma receptivity, and seed quality. Results showed that shading delayed flowering phenology, shortened the flowering stage, faded the flower colors, and significantly reduced pollen viability, stigma receptivity, the number of flowers, quantity, and quality of seeds. Under shading conditions, seed yield per plant was obviously positively correlated with germination potential, germination rate, pollen viability, and 1,000-seed weight. The number of flower buds, pollen viability, 1,000-seed weight, and germination rate had the greatest positive direct impact on seed yield per plant. Our findings suggested that delayed flowering and reducing reproduction growth were important strategies for alfalfa to cope with shading and pollen viability was the key bottleneck for the success of alfalfa reproduction under shading. However, given that alfalfa is a perennial vegetative-harvest forage, delaying flowering in a weak light environment was beneficial to maintain the high aboveground biomass of alfalfa. Therefore, this should be taken into account when breeding alfalfa cultivars suitable for intercropping. Future research should further reveal the genetic and molecular mechanism of delayed flowering regulating the accumulation and distribution of assimilates between vegetative and reproductive organs of alfalfa under shading, so as to provide a theoretical basis for breeding of shade-tolerant alfalfa cultivars.

## Introduction

Alfalfa (*Medicago sativa* L.) is a perennial forage with high quality and yield. As the most important legume forage in temperate regions, it is widely cultivated worldwide. Due to the rapid development of the dairy industry, China’s demand for alfalfa has increased significantly; therefore, alfalfa cultivation requires new niches, especially in southern China, where the land is mainly used for grain production and agriculture. The inter-row space of crops (such as maize and sorghum) or planted forests (such as orchard and poplar) provides opportunities for planting alfalfa. As a stress-resistant legume crop, alfalfa fixes a large amount of atmospheric nitrogen (approximately 135–605 kg ha^−1^ year^−1^; Putnam et al., 2001), which enables alfalfa to grow in various soil types and provide nitrogen for interplanting or subsequent crops when alfalfa is planted in interplanting or rotation system. Previous studies have shown that alfalfa has a certain tolerance to slight shade (about 50% of full sunlight; Lin et al., 1999; Qin et al., 2010) and can maintain high biomass. Therefore, as an interplanting crop, alfalfa plays an important role in the intercropping ecosystem. However, when the light intensity of the intercropping system is lower than 50% of full sunlight, the photosynthetic efficiency and morphogenesis of alfalfa have adaptive change, including obvious top advantage, thinner stems and easy to fall off, thinner leaves, fewer branches and lower relative growth rate, and delayed growth (collectively termed as shade avoidance symptoms, SAS), which significantly reduce the aboveground biomass of alfalfa (Cooper and Qualls, 1967; Walgenbach and Marten, 1981; Lin et al., 1999; Qin et al., 2010). However, the above characteristics are based on the observations of alfalfa seedlings, little is known about the reproduction of alfalfa under weak light stress.

Light is a key environmental factor that regulates plant growth and development. It is involved in controlling multiple responses in the plant life cycle, including seed germination, seedling de-etiolation, phototropism, shade avoidance, circadian rhythms, and flowering time (Castillon et al., 2007). Plants often grow in shaded environments, mainly from the shade of adjacent plant branches and upper canopy groups. As plants grow fixedly, they cannot choose appropriate living conditions through free movement like animals. Therefore, plants have developed a strict and precise light signal regulation system to regulate plant internal growth and metabolic activities, and produced corresponding reproductive strategies to maintain the persistence of their population in the shade environments (Boardman, 1977).

So far, plants have responded to shading through two flowering strategies. *Arabidopsis thaliana* (Cerdán and Chory, 2003), *Lotus japonicus* (Ueoka-Nakanishi et al., 2011), and rice showed early flowering induction in shade avoidance syndrome, which was regarded as a seed setting strategy before serious resources constraints (Carriedo et al., 2016). However, plants such as grape, tomato, and sunflower, when photon flux density decreased to approximately 40% of the full sunlight, flower bud differentiation was delayed, which lead to decrease in its distribution of accumulated dry matter to reproductive organs. In addition, the number of photosynthetic organs increased to adapt to weak light environments (El-Aidy et al., 1983; Atlag, 1990; El-Gizawy et al., 1993; Li et al., 2002). However, this strategy reduced flower bud quality, flowering rate, sex cell concentration (Yi et al., 2006), pollen viability, and ovum quality, thus increased dropping blossoms and fruit (Ferree et al., 1998), and finally reducing weight and composition of grains (Echarte et al., 2012). No matter which flowering strategy is adopted, it is the most suitable way for plants to survive in weak light environments.

In order to fully understand the shade tolerance potential in alfalfa, it is necessary to comprehensively study the reproductive strategy of alfalfa under shading conditions. Therefore, the objective of this study was to investigate the effects of artificial shade on flowering phenology, floral transition, flower development, fruiting, seed yield, and quality of two alfalfa cultivars planted for two consecutive years. The results of this study will helpful to reveal the mechanism of shading induced reproductive changes in alfalfa and provide a theoretical basis for screening and breeding of shade-tolerant and high-yielding cultivars in intercropping ecosystem.

## Materials and Methods

### Plant Materials and Growing and Shade Treatments

The experiment was conducted in three plastic greenhouses (4.5 m × 3 m × 2.5 m steel frame) from September 2017 to July 2018. Under natural photoperiod conditions (from winter 10 h/14 h to summer 14 h/10 h, day/night, during experiment period), there was no plastic cover within 50 cm from the ground, which was used for air circulation and free movements of pollinators (Supplementary Figure 1). The test site was located in Baima Teaching and Research Base of Nanjing Agricultural University (31°14′-32°37′N, 118°22′-119°14′E). The mean values of monthly day and night temperature outside the greenhouse during the flowering and fruiting period were shown in Supplementary Figure 2.

Two alfalfa cultivars Victoria (Fall Dormancy FD = 6) and Eureka (FD = 8) were selected for the experiment. The seeds of both cultivars were introduced from the United States. In September 2017, the seeds of each alfalfa cultivar were sowed in 24 separate pots (internal diameter 35 cm, height 27 cm) and filled with soil. The soil contained 90% humus soil and 10% pearlite (N + P + K > 4%, organic matter>30%), and 100 g organic fertilizer was applied in each pot. The seedlings grew continuously in three greenhouses, with eight pots in each greenhouse, and were thinned to five robust plants per pot when the seedlings were 4–5 cm (approximately 20 days after sowing).

In March 2018, after the alfalfa seedlings resumed growth from winter, two shading treatments and one full sunlight control treatment were set up. Two shading levels were created by using the neutral-density green shading net layer on the steel frame, and the light intensity (43.6 ± 1.3 and 21.3% ± 0.5% of full sunlight, marked as 56.4% shade and 78.7% shade, respectively) was measured by TES-1339 illuminance meter (Taiwan TES Electronic Industry Co., Ltd.). The experiment was conducted as completely random block design, with eight replicates for each treatment, and each pot was rotated to a new position every 14 days to reduce the influence of any position. Each pot was irrigated every 5 days to keep the soil moist. The shading treatment lasted for 5 months, covering the entire flowering, filling, and seed expansion season.

### Measurements of Flowering, Fruiting, and Seed Trait

#### Flowering Phenology

Three plants were randomly marked in each treatment pot, and the first flowering date of each plant was record. The floral buds of marked plants were counted every 3 days, with 50% of floral buds in the bud initiation stage as the budding stage, the flowering stage of 10% of the alfalfa plants in flowering stage as the early flowering stage, and 80% of the alfalfa plants in the flowering stage as the full flowering stage (Mao, 2019). Under each shading treatment, the flowering stage was recorded from the first appearance of flower to the withering of the last flower.

#### Flowering Dynamics

Alfalfa is a cross-pollinated forage crop. In the experimental area, its flowering stage is from April to July, and the main pollinators are bees, especially *Megachile rotundata Fubricius*. During this period, at each light level, three plants per pot were randomly selected from each pot under shading treatment. The buds and flowers in three inflorescences of each plant were counted every day from the first appearance of the buds to the end of the flowering stage. The number of fallen flowers was estimated by counting the drop mark of the flowering peduncles. The following formula was used to calculate the flowering rates and fallen flower rates.


(1) $$\mathrm{Flowering rate}\;\left(\%\right)=\left(\begin{array}{l} \mathrm{Number of the flowering buds}/\\ \mathrm{Number of buds} \end{array}\right)\times 100\%$$

(2) Fallen flower rate%=Number of the drop mark of peduncles/Number of flowers×100%

#### Pollen Viability and Stigma Receptivity

Pollen fertility and stigma receptivity were tested by potassium iodide method and benzidine hydrogen peroxide method (Dafni and Maués, 1998), respectively. At each shading treatment, three plants were randomly selected from each pot. During the full flowering stage (May to June), three inflorescences of each plant at the same flowering stage were marked. In the morning, the pollen grains of five buds in each inflorescence were collected by gently peeling the keels in Petri plates and transferred to glass slides. The pollen grains were stained with 0.5% iodine and 1% potassium iodide solutions, and observed and counted under a microscope at ×10 magnification. The stigmas of the five flowers of each plant were immersed in the groove of concave glass slide with benzidine hydrogen peroxide (1% benzidine:3% hydrogen peroxide:H_2_O = 4:11:22, volume ratio). More than two-thirds of the stigma turned blue, and when many bubbles formed around the stigmas, which indicated that the stigmas were receptive. The test lasted for 5 days, and the flowers were tagged at different days after flowering. The following formula was used to calculate pollen viability and stigma receptivity:


(3) $$\mathrm{Pollen viability}\;\left(\%\right)=\left(\begin{array}{l} \mathrm{Number of blue pollens}/\\ \mathrm{Number of pollens} \end{array}\right)\times 100\%$$

(4) $$\mathrm{Stigma receptivity}\;\left(\%\right)=\left(\begin{array}{l} \mathrm{Number of blue stigmas}/\\ \mathrm{Number of stigmas} \end{array}\right)\times 100\%$$

#### Seed Production and Quality

Three plants were randomly selected from each pot under shading treatment. The pods in the three inflorescences of marked plants were counted every day from the first appearance of the pods to the end of the seed production. The number of fallen pods was estimated by counting the drop marks of the pods every day. After collection, the total seed yield of each plant was determined and the seeds were air dried. The seeds produced by each plant were counted, and then, the 1,000-seed weight was determined in four replications. Germination study was conducted in a Petri plate, in which 100 seeds were placed on the top of a wet germination filter paper and stored in a germinator at 20°C ± 2°C. The first count on the 4th day, and the second count on the 10th day. The seedlings with roots longer than seed and at least one cotyledon connected to the root as germinating seeds.

The following formulas were used to calculate the pod setting rate, pod falling rate, germination potential, and germination rate:


(5) $$\mathrm{Pod}\;\mathrm{setting rate}\;\left(\%\right)=\left(\begin{array}{l} \mathrm{Number of pods}/\\ \mathrm{Number of flowering buds} \end{array}\right)\times 100\%$$

(6) Podfalling rate%=Number of the drop mark ofpod/Number of pods×100%

(7) Germination potential%=Number of germinated seedsonthe 4thday/Number of sampled seeds×100%

(8) Germination rate%=Number of germinated seedsonthe10thday/Number of sampled seeds×100%

### Statistical Analysis

The differences of plant morphological and physiological variables under shading treatment were determined by using one-way ANOVA followed by Duncan new multiple range test (95% CI), and the standard errors of the arithmetic means were provided. The correlation and path analysis of seed yield per plant and flowering and fruiting factors were carried out by using Excel 2016 and SPSS 16.0. Pollen viability and average stigma receptivity within 5 days were used in the two analyses.

## Results

### Flowering Phenology

Compared with alfalfa without shading treatment, alfalfa under shade entered budding stage, early flowering stage to full flowering stage later and had a shorter flowering stage. Under different shading levels, the flowering phenology and change trend of the two alfalfa cultivars were similar (Table 1). The budding stage of plants under 56.4% shade was delayed by 2–4 days, and the budding stage under 78.7% shade was delayed by 13–16 days. The early flowering stage of shaded plants delayed 2–4 days under 56.4% shade and 14–16 days under 78.7% shade, respectively. The full flowering stage of shaded plants delayed 5–8 days under 56.4% shade and 8–11 days under 78.7% shade. Moreover, the flowering stage of alfalfa was 115–120 days under full sunlight treatment, 67–69 days under 56.4% shade treatment, and 48–49 days under 78.7% shade treatment.

**Table 1:** Flowering phenology of two alfalfa cultivars under different shading levels.

Parameters	Treatment	Cultivar	
		Victoria	Eureka
Budding stage	Full light	April 18th	April 22th
	56.4% shade	April 22th	April 24th
	78.7% shade	May 3rd	May 4th
Early flowering stage	Full light	May 4th	May 6th
	56.4% shade	May 8th	May 8th
	78.7% shade	May 20th	May 20th
Full flowering stage	Full light	May 12th	May 17th
	56.4% shade	May 20th	May 22th
	78.7% shade	May 31st	May 30th
Flowering stage	Full light	April 18–August 17 (lasted 123 days)	April 22–August 17 (lasted 119 days)
	56.4% shade	April 22–July 1 (lasted 72 days)	April 24–July 1 (lasted 70 days)
	78.7% shade	May 3–June 21 (lasted 49 days)	May 4–June 23 (lasted 50 days)

### Flower Colors

**Figure 1:**
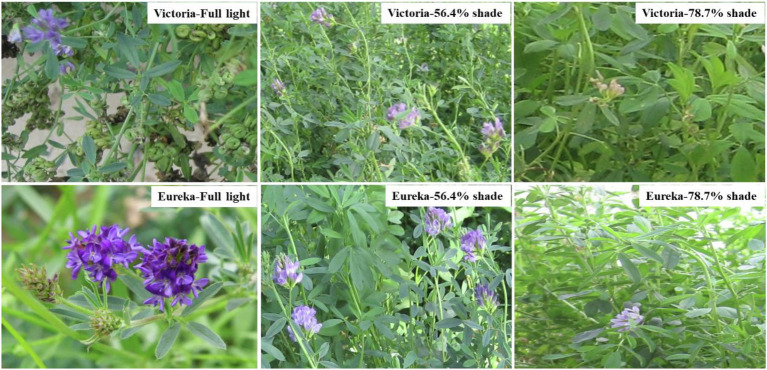
Flower color of two alfalfa cultivars under different shading levels.

The color of alfalfa plant under shading treatment was lighter, and there were fewer inflorescences and flowers per inflorescence (Figure 1). With the increase of shading, the flower color of the two alfalfa cultivars gradually faded, that is, the flower color of full sunlight treatment was dark purple, 56.4% shade was medium purple, and 78.7% shade was light purple.

### Dynamics of Pollen Viability and Stigma Receptivity

**Figure 2:**
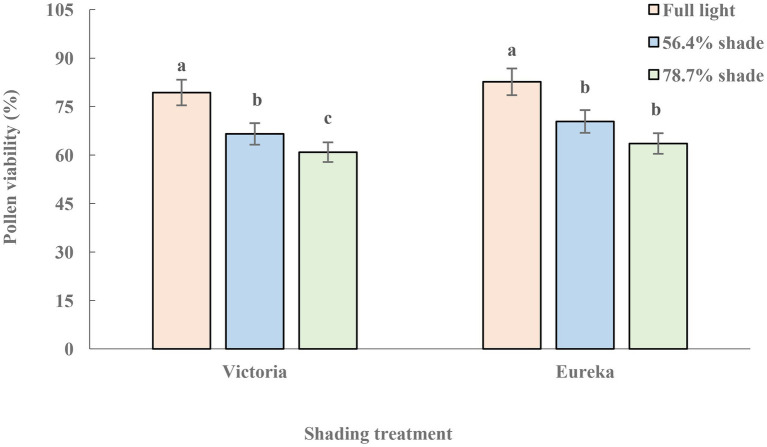
Pollen viability of two alfalfa cultivars under different shading levels. Different letters indicate significant differences (*p <* 0.05).

The pollen viability of two alfalfa cultivars decreased significantly with increase of shade (*p* < 0.05), and the decline of Victoria was greater than that of Eureka (Figure 2).

**Figure 3:**
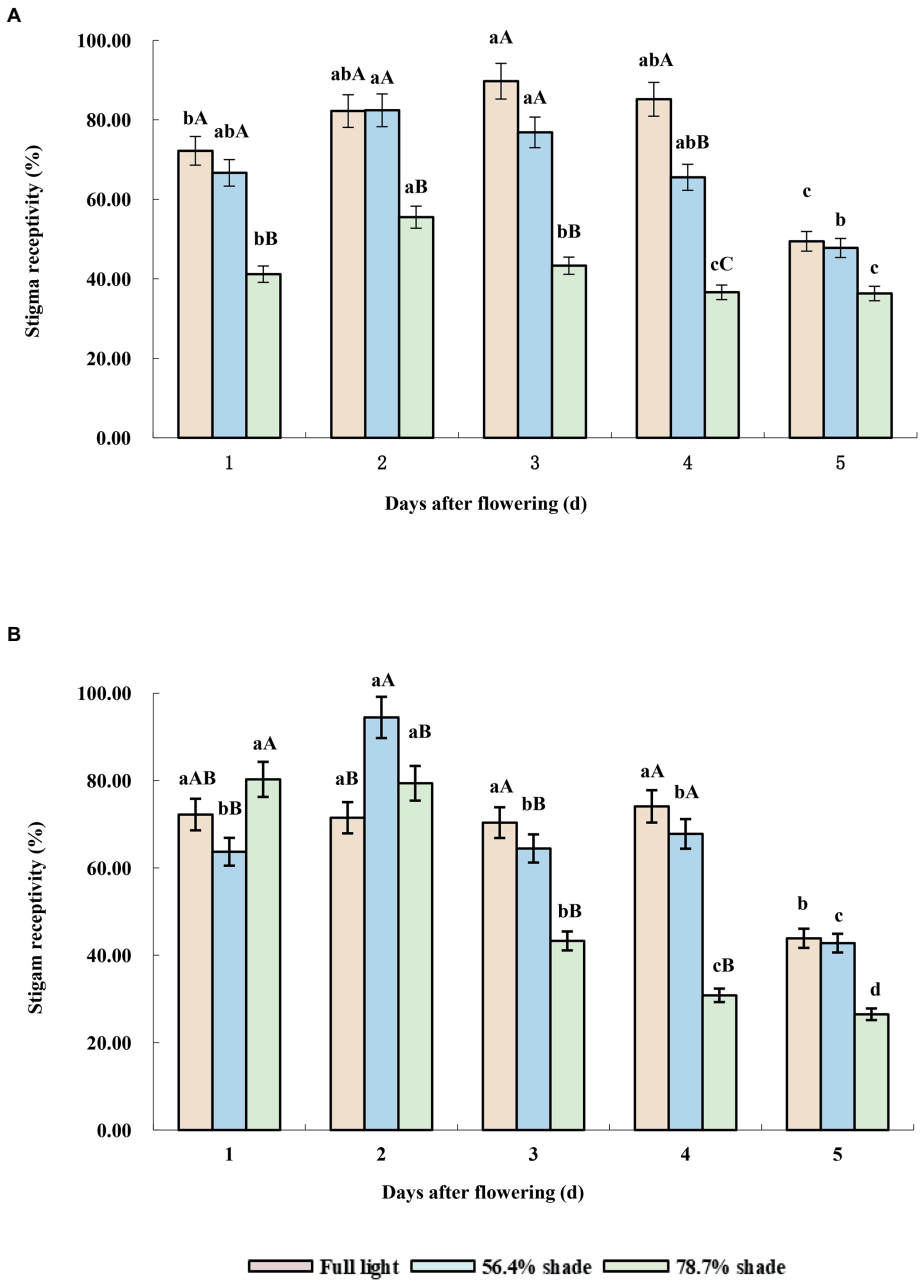
Stigma receptivity of the two alfalfa cultivars under different shading levels. **(A)** Victoria, **(B)** Eureka. Different capital and lower case letters indicate significant difference under different shading levels and days after flowering at 0.05 level (*p <* 0.05), respectively.

Under full sunlight treatment, the stigma receptivity of the two cultivars was higher, from the first to the fourth day after flowering, but decreased significantly from the fifth day after flowering. Under 56.4% shade treatment, the stigma receptivity of the two cultivars was higher in the first 2 days after flowering, decreased gradually from the third day of flowering, and decreased significantly on the fifth day of flowering (*p* < 0.05). Under 78.7% shade treatment, the stigma receptivity of the two cultivars decreased significantly from the third days after flowering, but their response to shade was different in the first 2 days after flowering. The stigma receptivity of Eureka was not significantly different from that of full sunlight and 56.4% shading treatment, while that of Victoria was significantly lower than that of full sunlight and 56.4% shading treatment, which showed that shade had a great effect on pollen viability and sigma receptivity in Victoria (Figures 3A,B).

### Flowering, Fruiting, and Seed Traits

**Figure 4:**
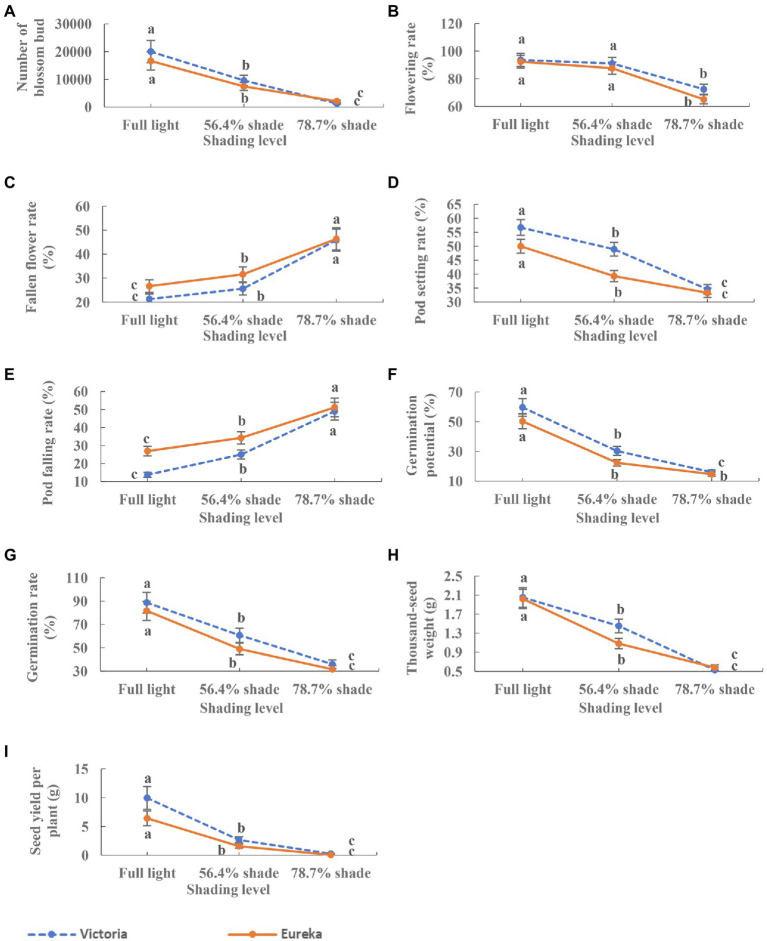
Dynamics of flowering, fruiting, and seed traits of two alfalfa cultivars under different shading levels. **(A)** Number of flower bud, **(B)** flowering rate, **(C)** fallen flower rate, **(D)** pod setting rate, **(E)** pod falling rate, **(F)** germination potential, **(G)** germination rate, **(H)** 1,000-seed weight, and **(I)** seed yield per plant. Different low case letters indicate significant differences under different shading levels at 0.05 level (*p <* 0.05), respectively.

Shade significantly inhibited the flowering and fruiting of two alfalfa cultivars. With the increase of shade, the number of flower bud, flowering rate, and pod setting rate decreased significantly (*p <* 0.0001; Figures 4A,B,D), while the rate of flower and pod falling rate increased significantly (*p* < 0.0001; Figures 4C,E). Seed germination potential, germination rate, 1,000-seed weight, and seed yield per plant decreased significantly with the increase of shade (*p* < 0.0001; Figures 4F–I).

### Correlation Analysis Between Seed Yield per Plant and Flowering and Fruiting Factors

The seed yield per plant of Victoria was significantly positively correlated with germination potential, pollen viability, and 1,000-seed weight (Table 2). The seed yield per plant of Eureka was significantly positively correlated with the germination potential, germination rate, and pollen viability (Table 3).

**Table 2:** Correlation between the seed yield per plant and flowering and fruiting factors of Victoria.

Trait	y	x1	x2	x3	x4	x5	x6	x7	x8	x9	x10
y	1										
x1	0.988	1									
x2	0.746	0.840	1								
x3	−0.779	−0.866	−0.999^*^	1							
x4	0.885	0.946	0.971	−0.982	1						
x5	−0.867	−0.933	−0.979	0.988	−0.999^*^	1					
x6	0.998^*^	0.996	0.791	−0.821	0.915	−0.920	1				
x7	0.989	1.000^**^	0.838	−0.864	0.944	−0.931	0.997^*^	1			
x8	0.999^*^	0.995	0.78	−0.811	0.908	−0.926	1.000^**^	0.995	1		
x9	0.805	0.887	0.996	−0.999^*^	0.989	−0.916	0.844	0.884	0.834	1	
x10	0.998^*^	0.996	0.787	0.818	0.913	−0.997^*^	1.000^**^	0.996	1.000^**^	0.841	1

y, seed yield per plant; x1, number of flower bud; x2, flowering rate (%); x3, fallen flower rate (%); x4, pod setting rate (%); x5, pod falling rate (%); x6, germination potential (%); x7, germination rate (%); x8, pollen viability (%); x9, average stigma receptivity (%); and x10, 1,000-seed weight (g).

* Respective correlation is significant at the 0.05 level.

** Respective correlation is extremely significant at the 0.01 level (two tailed).

**Table 3:** Correlation between seed yield per plant and flowering and fruiting factors of Eureka.

Trait	y	x1	x2	x3	x4	x5	x6	x7	x8	x9	x10
y	1										
x1	0.990	1									
x2	0.791	0.870	1								
x3	−0.837	−0.907	−0.997^*^	1							
x4	0.991	0.997^*^	0.831	−0.873	1						
x5	−0.867	−0.929	−0.991	0.998^*^	−0.899	1					
x6	1.000^**^	0.987	0.778	−0.826	0.996	−0.856	1				
x7	0.997^*^	0.998^*^	0.836	−0.877	1.000^**^	−0.903	0.995	1			
x8	0.997^*^	0.998^*^	0.835	−0.876	1.000^**^	−0.902	0.995	1.000^**^	1		
x9	0.672	0.771	0.985	−0.968	0.721	−0.952-	0.656	0.727	0.726	1	
x10	0.992	1.000^**^	0.860	−0.898	0.999^**^	−0.921	0.990	0.999^*^	0.999^*^	0.757	1

y, seed yield per plant; x1, number of flower bud; x2, flowering rate (%); x3, fallen flower rate (%); x4, pod setting rate (%); x5, pod falling rate (%); x6, germination potential (%); x7, germination rate (%); x8, pollen viability (%); x9, average stigma receptivity (%); and x10, 1,000-seed weight (g).

* Respective correlation is significant at the 0.05 level.

** Respective correlation is extremely significant at the 0.01 level (two tailed).

### Path Analysis Between Seed Yield per Plant and Flowering and Fruiting Factors

Path analysis results showed that there were differences in the effects of flowering and fruiting factors on seed yield per plant between the two alfalfa cultivars.

In terms of flowering factors, the direct path coefficient of Victoria flower bud number on seed yield per plant was the highest (0.905), while the direct path coefficient of average stigma receptivity on seed yield per plant was the lowest (−0.552). The direct path coefficient of pollen viability was very low (0.390), but it had higher indirect path coefficients with flower bud number (0.901) and flowering rate (0.460), which indirectly affected the seed yield per plant, so it had a significant positive correlation (0.999) with the seed yield per plant. The direct path coefficient of Eureka pollen viability on seed yield per plant was the highest (0.986), but the direct path coefficients of flowering rate, fallen flower rate, and average stigma receptivity on seed yield per plant were lower, which were −0.203, −0.171, and −0.075, respectively (Table 4).

**Table 4:** Path analysis of seed yield per plant and flowering factors of two alfalfa cultivars.

Cultivar	Trait	Correlation coefficient	Direct effect	Indirect effect (r_ij_ → y)				
				x_i1_ → y	x_i2_ → y	x_i3_ → y	x_i4_ → y	x_i5_ → y
Victoria	x1	0.988	0.905		0.496	−0.311	0.388	−0.490
	x2	0.746	0.590	0.760		−0.358	0.304	−0.550
	x3	−0.779	0.359	−0.784	−0.590		−0.316	0.552
	x4	0.999	0.390	0.901	0.460	−0.291		−0.460
	x5	0.805	−0.552	0.803	0.588	−0.358	0.325	
Eureka	x1	0.990	0.086		−0.177	0.155	0.984	−0.058
	x2	0.791	−0.203	0.074		0.170	0.823	−0.074
	x3	−0.837	−0.171	−0.078	0.202		−0.864	0.073
	x4	0.997	0.986	0.085	−0.169	0.150		−0.055
	x5	0.672	−0.075	0.066	−0.200	0.165	0.716	

y, seed yield per plant; x1, number of flower bud; x2, flowering rate; x3, fallen flower rate; x4, pollen viability; and x5, average stigma receptivity.

In terms of fruiting factors, the direct path coefficient of Victoria 1,000-seed weight to seed yield per plant was the highest (0.669), and the direct path coefficient of pod setting rate was the lowest (−0.279). The direct path coefficient of germination potential on seed yield per plant was low (0.057), but it had a greater indirect effect (0.519), the indirect coefficient of 1,000-seed weight was higher (0.669), and there was a significant positive correlation with seed yield per plant (0.998). The direct path coefficient of Eureka germination rate on seed yield per plant was the highest (1.085), and the direct path coefficient of pod setting rate was the lowest (−1.145). The direct path coefficient of germination potential was lower, but the indirect coefficient with pod falling rate (0.357), germination rate (1.079), and 1,000-seed weight (0.767) was higher, which could have a great indirect effect on seed yield per plant, and had a very significant positive correlation (1.000) with seed yield per plant (Table 5).

**Table 5:** Path analysis of seed yield per plant and fruiting factors of two alfalfa cultivars.

Cultivar	Trait	Correlation coefficient	Direct effect	Indirect effect (r_ij_ → y)				
				x_i6_ → y	x_i7_ → y	x_i8_ → y	x_i9_ → y	x_i10_ → y
Victoria	x6	0.885	−0.279		0.005	0.052	0.492	0.611
	x7	−0.867	−0.005	0.279		−0.051	−0.485	−0.600
	x 8	0.998	0.057	−0.255	0.005		0.519	0.669
	x 9	0.989	0.521	−0.263	0.005	0.994		0.667
	x10	0.998	0.669	−0.255	0.005	0.057	0.519	
Eureka	x6	0.991	−1.145		−0.375	0.295	1.085	0.774
	x7	−0.867	−0.418	1.029		−0.254	−0.979	−0.714
	x 8	1.000	0.296	−1.140	0.357		1.079	0.767
	x 9	0.997	1.085	−1.145	0.377	0.295		0.774
	x10	0.992	0.775	−1.144	0.385	0.293	1.083	

y, seed yield per plant; x6, pod setting rate; x7, pod falling rate; x8, germination potential; x9, germination rate; and x10, 1,000-seed weight.

## Discussion

As mentioned above, delayed flowering was one of important adaptive strategies of plant in response to shade. However, how plants adjust their flowering time to adapt to weak light has not been tested so far. Previous studies on *Antirrhinum majus* (Munir et al., 2004), bedding ornamentals (Faust et al., 2005), *Swarnaprabha* rice (Panigrahy et al., 2019), and some soybean lines (Cober and Voldeng, 2001) showed that shading led to delayed flowering, and the reduction of carbohydrate availability under shading was partly responsible for the delayed flowering (Bernier et al., 1993). The addition of sucrose to flowering *Arabidopsis* grown in the dark proved that carbohydrate reserve can replace the current photosynthesis during floral induction (Roldan et al., 1999). Moreover, the weakening of photosynthesis, photosynthate accumulation, and reproductive growth in competition for photosynthetic products under shading delayed flower bud differentiation, reduced inflorescence and flowers, and shortened flowering time (Cober and Voldeng, 2001; Panigrahy et al., 2019). This was consistent with the results of alfalfa. Previous studies showed that compared with other cultivars, shade tolerant cultivar Victoria had less reduction in biomass (including leaf, stem and individual biomass), crown branch number and leaf anatomical structure (including parenchyma thickness, palisade cell width, mesophyll thickness and midvein thickness) under shading conditions (Qin et al., 2010, 2012, 2014). Moreover, recent finding showed the increased expression of miR156 reduced the transcription level of its targeted gene *msSPL_3_*, thus prolonging the growth period of vegetative stage and delaying the flowering time in alfalfa under shading (Aung et al., 2015; Lorenzo et al., 2019). It showed delayed flowering under weak light condition was conducive to the maintenance of aboveground biomass of alfalfa to a certain extent. However, as a perennial forage, the effect of delayed flowering on the accumulation, distribution of assimilates, and survival of alfalfa under shading needed to be monitored and researched.

Alfalfa is a monoecious, entomophilous, and cross-pollinated plant. When the flower bud develops to the fourth stage, the anther has burst and covered the stigma. At this time, almost all the mature pollen has been scattered on the stigma. However, without insect pollination, the seed setting rate was still very low (Jiang et al., 2003). It was reported that the color and size of flowers were the primary factors to attract bees, bright colors can attract insects to spread pollen and promote plant reproduction, while faded or withered flowers reduced the access of bees, thus reducing seeds yield in a weak light environment (Clement, 1965). Anthocyanin is an important pigment in flower color and its biosynthesis, accumulation, and metabolic regulation were induced by light (Zhao et al., 2012). Under shading conditions, the insufficient supply of soluble sugars, such as sucrose, significantly decreased the content of anthocyanin in petals and inhibited the biosynthesis of anthocyanin in petals. In addition, molecular biology studies showed that the decline of anthocyanins in shade plants was the result of the joint action of all anthocyanin biosynthetic genes, especially the upstream *PlPAL* and *PlCHS*, whose expression decreased significantly under weak light and the upstream substrate inducing anthocyanin synthesis decreased significantly (Stein and Hensen, 2013). In order to clarify the effect of fading flower on seed setting rate of alfalfa, it is necessary to further study the visiting behave of insect in alfalfa under shade.

In the process of plant sexual reproduction, pollination begins with flower bud differentiation, anther dehiscence, and mature pollen release. Pollen grains carrying live male gametes or precursors must reach appropriate receptive stigmas for smooth fertilization (Yi et al., 2006). Therefore, pollen viability and stigma receptivity were the key steps for successful plant reproduction. Shading can inhibit the photosynthesis, lead to pistil atrophy, reduce the number of pollen, inhibit pollen germination and pollen tube growth, and increase ratio of abnormal pollen (Alice, 1996). These all led to the decline of alfalfa pollen vitality (Figure 2). It was worth mentioning that shading reduced the nutrient richness of pollen and nectar, which were the most attractive part of plants to insects. Nectar can absorb ultraviolet spectrum and can be accurately detected by insects. The pattern and spectral range of nectar reflected fluorescence provide pollinating insects with information on nectar existence and abundance (Jiang et al., 2003). Therefore, the decrease of energy, pollen, and nectar concentration may be an important factor affecting the successful pollination of alfalfa under shading conditions. The shortening of stigma receptivity duration under shading (Figure 3) may be due to the decrease of sex cells concentration (Yi et al., 2006) and stigma peroxidase (POD) activity, which was significantly positively correlated with light intensity (Sui et al., 2005). So far, there are few studies on the effects of shading on plant pollen viability and stigma receptivity, and its mechanism needs to be further studied.

The seed yield per plant of alfalfa depended on the number of flowers per branch, the number of florets per inflorescence, the number of ovules per floret, the number of pods per branch, the number of seeds per pod, and 1,000-seed weight, which all change with environmental conditions (Chen, 2001). Among the environmental factors, incident or intercepted solar radiation had proved to be the most influential (Izquierdo et al., 2008). Long-term shading reduced the flower bud yield, flowering rate, and pod setting rate of alfalfa, and increased fallen flower and fallen pod rates (Figure 4). These may be due to the insufficient supply of photo-assimilates and minerals. In the key early stage of development, the competition of stem growth for assimilates was better than that of fruit growth (Islam et al., 2005). This lead to a decline in pollen viability and fertilized eggs quality, as well as a decline in flowers and fruits under shading (Bepetel and Lakso, 1998). In this process, weak light reduced the production of flowering-related hormones, such as gibberellic acid, which can promote flower stem elongation and prevent fruit abscission, plays a key role (Folta and Maruhnich, 2007; Fukuda, 2013). From the beginning of seed filling to full maturity, weak light stress can lead to seed deterioration and reduce seed viability and vigor by changing seed components (Cockshull and Graves, 1992). It is reported that shading increases oil content, linoleic acid, and linolenic acid (Bellaloui et al., 2012), which limited C and N assimilation, thus reducing nitrate reductase activity, chlorophyll concentration, seed protein, and oil accumulation (Allen et al., 2009; Board et al., 2010). In this study, the effects of shading on alfalfa germination potential, germination rate, and 1,000-seed weight may have a similar mechanism. However, further research was needed to explain this possibility.

The 1,000-seed weight and germination rate of seeds indicated the size, plumpness, and vitality of seeds, which were determined by the assimilates allocated to reproductive organs. In this study, the two factors that contributed the most to the seed yield per plant showed that the main reason for the decline of seed yield per plant was the reduction of assimilates allocated to seeds under weak light environments (Table 5). It should be noted that the pod setting rate had the greatest negative direct impact on the seed yield per plant and greatest negative indirect impact on germination potential, germination rate, and 1,000-seed weight (Table 5). The data available so far suggested that the weight and composition of seeds are the results of a complex process, among which vegetative organs (leaves) promote the supply of assimilates to the seeds, while reproductive structures (pods and seeds) may locally regulate seed metabolism (Bianculli et al., 2016). It is reported that compared with leaves, the soybean pods have contributed to the low-carbon economy under shading (Allen et al., 2009). In this work, it was difficult to assess whether this effect was due to the contribution of pods and leaves in transporting assimilates to seeds or to the competition of assimilates between pods and leaf synthesis. In order to clarify this point, it is necessary to further study the transport and allocation dynamics of the assimilates between alfalfa leaves and pods under weak light conditions.

Weak light had harmful to flowering, fruiting, and seed production of alfalfa. The extremely low seed yield per plant under shade showed that the reproductive growth of alfalfa was extremely dependent on light intensity, and it is a species with high light demand. Delayed flowering time, shortened flowering stage, decreased quantity and quality of flowers and fruits, decreased pollen viability and stigma receptivity, indicating that reducing the distribution of assimilates to reproduction growth was an important strategy for alfalfa to response to shading. However, because alfalfa is a perennial vegetative-harvest forage, this strategy may be beneficial to maintain the high aboveground biomass of alfalfa under weak light environment. Therefore, this strategy should be considered when breeding shade-tolerant cultivar under intercropping system. Future research should further reveal the genetic and molecular mechanism of delayed flowering regulating the accumulation and distribution of assimilates between vegetative and reproductive organs of alfalfa under shading, so as to provide a theoretical basis for breeding shade-tolerant alfalfa cultivars.

## Data Availability Statement

The raw data supporting the conclusions of this article will be made available by the authors, without undue reservation.

## Author Contributions

FQ and HQ conceived and designed the research. FQ, GT, HL, and ZC performed the experiments and data analysis. FQ and HQ analyzed the data and wrote the manuscript. ZL, YS, JF, and LK advised on the results and discussions. All authors discussed the results and implications, commented on the manuscript at all stages, and contributed to the article and approved the submitted version.

## Funding

This work was financially supported by the National Key R&D Program of China, grant/award number: 2017YFC0506005; Open Innovation and Entrepreneurship Training Program of National Experimental Teaching Demonstration Center of Plant Production of Nanjing Agricultural University: ZKF202106; and SRT program of Nanjing Agricultural University: 202126XX04.

## Conflict of Interest

The authors declare that the research was conducted in the absence of any commercial or financial relationships that could be construed as a potential conflict of interest.

## Publisher’s Note

All claims expressed in this article are solely those of the authors and do not necessarily represent those of their affiliated organizations, or those of the publisher, the editors and the reviewers. Any product that may be evaluated in this article, or claim that may be made by its manufacturer, is not guaranteed or endorsed by the publisher.

## Supplementary Material

The Supplementary Material for this article can be found online at: https://www.frontiersin.org/articles/10.3389/fpls.2022.835380/full#supplementary-material
