# Supplementary figures and images for: Shade Delayed Flowering Phenology and Decreased Reproductive Growth of Medicago sativa L
Source: Front Plant Sci. 2022 Jun 2;13:835380. doi: 10.3389/fpls.2022.835380 (PMC9203126; doi:10.3389/fpls.2022.835380)

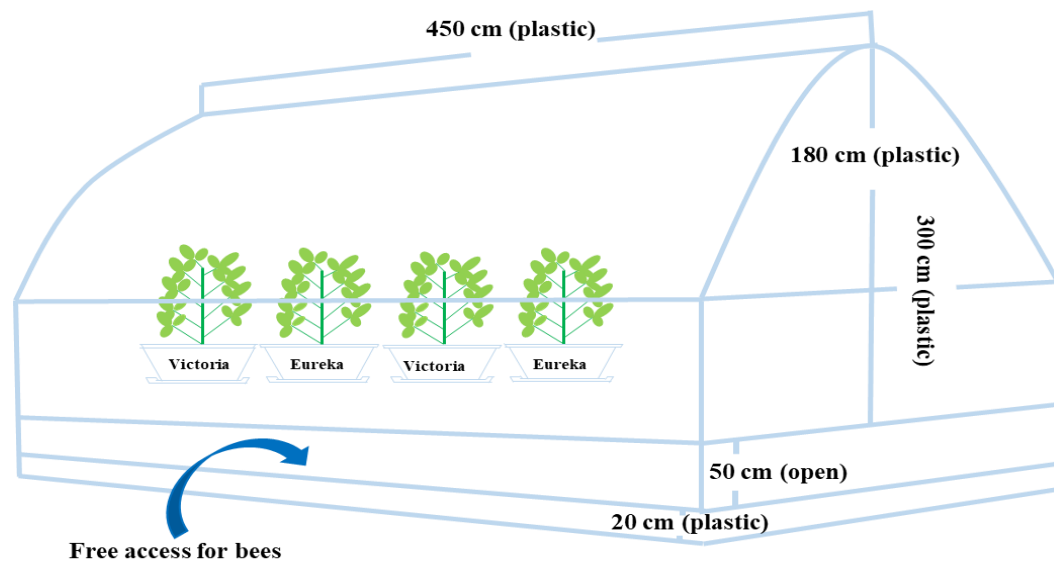

**FIGURE S1** | Diagram of the plastic greenhouse.

Supplement: Supplementary file 1 [file Image_1.pdf]
