# Supplementary material for: Shade Delayed Flowering Phenology and Decreased Reproductive Growth of Medicago sativa L
Source: Front Plant Sci. 2022 Jun 2;13:835380. doi: 10.3389/fpls.2022.835380 (PMC9203126; doi:10.3389/fpls.2022.835380)
Supplement: Supplementary file 2 [file Image_2.pdf]

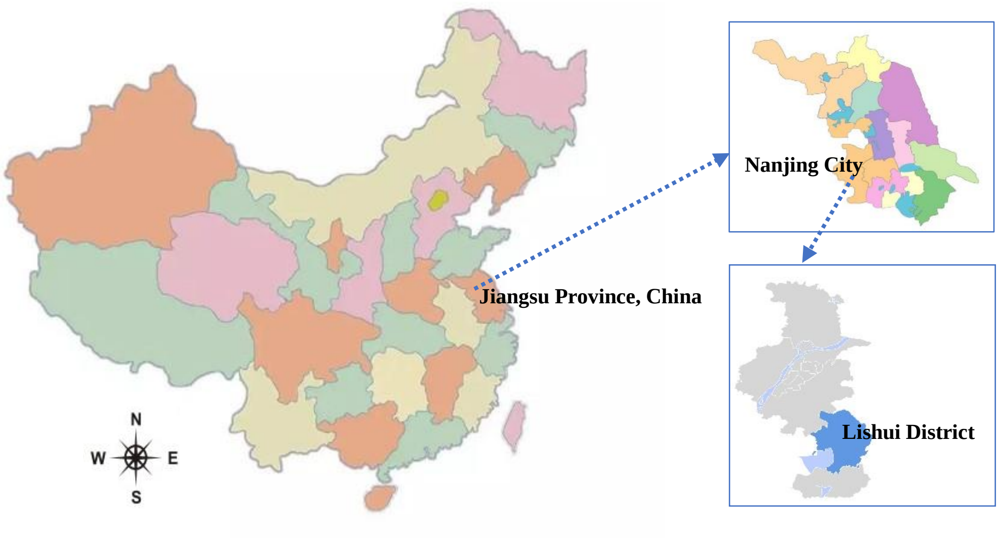

Item	Month									
	March		April		May		June		July	
	Day	Night	Day	Night	Day	Night	Day	Night	Day	Night
**Maximum temperature (℃)**	20.00	10.00	28.00	16.00	34.00	20.00	35.00	25.00	36.00	27.00
**Minimum temperature (℃)**	7.00	−1.00	13.00	3.00	21.00	13.00	24.00	19.00	27.00	22.00
**Mean temperature (℃)**	13.32	3.65	21.51	10.22	27.64	17.13	30.51	22.32	32.73	25.40

FIGURE S2 | Experimental area and temperatures outside the testing green house from March to July of 2018.
